# Health professionals’ experiences of providing care for women survivors of sexual violence in psychiatric inpatient units

**DOI:** 10.1186/s12913-019-4683-z

**Published:** 2019-11-14

**Authors:** Carol O’Dwyer, Laura Tarzia, Sabin Fernbacher, Kelsey Hegarty

**Affiliations:** 1 Department of General Practice, Melbourne Medical School, Faculty of Medicine, Dentistry & Health Sciences, The University of Melbourne, Level 2, 780 Elizabeth Street, Melbourne, Victoria 3010 Australia; 2 Centre for Family Violence Prevention, The Royal Women’s Hospital, The University of Melbourne, Melbourne, Victoria 3052 Australia; 3 The Royal Women’s Hospital, Department of General Practice | Melbourne Medical School, Faculty of Medicine, Dentistry & Health Sciences, The University of Melbourne, Level 2, 780 Elizabeth Street, Melbourne, Victoria 3010 Australia; 4 Consultant, Melbourne, Australia

**Keywords:** Australia, Sexual violence, Mental health, Women, Health professionals, Psychiatric inpatient unit, Qualitative

## Abstract

**Background:**

Survivors of sexual violence, who are predominantly women, commonly access mental health services. Psychiatric inpatient units in Australia are predominately mixed gender and may further retraumatise these women. Sexual violence is under-recognised by mental health professionals and there is a lack of adequate policy or direction for mental health service services. To date, only a small amount of research has focused on health professionals’ experiences of providing trauma-informed care to women in psychiatric settings, with most studies focused on specific practices or interventions. Qualitative data is particularly lacking on this topic. This is a critical gap in the knowledge given that health professionals are key to detecting and addressing victimisation. The aim of this study was to gain an in-depth understanding of healthcare professionals’ experiences and perceptions in providing care to women who are survivors of sexual violence in psychiatric inpatient units.

**Methods:**

This qualitative study utilised semi-structured interviews with 40 health professionals recruited from four psychiatric inpatient units within a large Australian public mental health organisation. Data were examined using thematic analysis.

**Results:**

Three main typologies were developed to describe participants’ experiences of the care provided to women; 1) *Dismissing and denying; 2) Acknowledging but unprepared; 3) Empathising but despairing.*

**Discussion:**

Gender, professional training, adherence to the biomedical model, and level of experience influenced health professionals’ experiences.

**Conclusions:**

Health professionals in this study held varying attitudes towards female consumers and responses to sexual violence. Our findings suggest the need to address individual staff perception and promote trauma-informed and gender-sensitive care across all disciplines, genders, and levels of experience.

## Introduction

Sexual violence is prevalent in Australia and worldwide [1, 2] and it impacts negatively on mental health [2]. The World Health Organisation defines sexual violence as “any sexual act, attempt to obtain a sexual act, unwanted sexual comments or advances, or acts to traffic or otherwise directed against a person’s sexuality using coercion, by any person regardless of their relationship to the victim, in any setting, including but not limited to home and work” ([3] p. 149). In Australia one in five women experience sexual violence, usually perpetrated by someone known to them including an intimate partner [1]. Sexual violence is associated with morbidity and mortality; including substance misuse, mental illness and suicidal behavior [4]. The relationship between sexual violence and mental health is complex and bi-directional [5]. Research shows that sexual violence predisposes and maintains mental illness [2], conversely severe mental illness can increase women’s risk of experiencing sexual violence [4].

Sexual violence is common in women who access mental health services. One in three women presenting at inpatient or outpatient mental health services has previously experienced domestic violence, including sexual violence [6]. The prevalence of sexual violence reported at time of admission to a psychiatric inpatient unit in Australia and USA varies across studies, with estimates between 5 and 45% [3]. A small study in Victoria (*n* = 50), Australia noted that almost half (45%) of women reported historic sexual assault and 67% reported sexual or other harassment while accessing a psychiatric inpatient unit [7].

Sexual violence is a highly gendered issue [3, 7]. In Australia, most psychiatric units have mixed wards [8] with two-thirds of women inpatients reportedly feeling unsafe in this environment and experiencing harassment, intimidation or abuse [9]. Similarly, in the UK over a third of psychiatric inpatients reported being attacked, threatened or made to feel unsafe [10]. Encouragingly, women with mental illness are more likely to disclose sexual violence to health professionals (including primary and secondary health care services) than to informal supports [11].

Studies indicate that mental health professionals are cognisant of the need to appropriately respond to women who have experienced sexual violence in a gender-sensitive and safe manner [11]. However, sexual violence is generally not identified by mental health services [12]. Staff frequently do not ask about a history of sexual violence due to lack of knowledge, confidence and skills to respond appropriately to such disclosures [13–15]. Furthermore, although mental health policies acknowledge the connection between sexual violence and mental illness, they lack direction or description of recommended action for mental health services and clinical care [16]. These practices are linked to the current dominant model of care in psychiatric settings that focuses on the biological symptoms of mental illness rather than the psychosocial [17]. Trauma-informed care is a model of care that recognizes the responsibility of mental health services to be responsive to trauma at a systemic level [18–20]. It aims to look through a ‘trauma lens’ to improve experiences, relationships and environments for consumers and health professionals [21].

There is limited research examining how healthcare professionals understand and experience providing care to women with a history of sexual violence and mental illness in psychiatric inpatient units. This knowledge is integral to the provision of high quality, gender-sensitive care and for ensuring the safety of consumers during their admission.

### Study aim

The aim of this study is to gain an in-depth understanding of healthcare professionals’ experiences and perceptions in providing care for women in psychiatric inpatient units who are survivors of sexual violence.

## Methods

All participants have been provided with a pseudonym for de-identification purposes. This qualitative study involved semi-structured interviews within four different psychiatric inpatient units within one mental health organization in Victoria, Australia. The study specifically focused on health professionals’ perceptions of care provided to women who are survivors of sexual violence. In Australia, the term ‘consumer’ is commonly used for people with a mental illness who access mental health services. The term ‘female consumer’ is used throughout this article to describe women accessing mental health services who are also survivors of sexual violence.

### Recruitment

A purposive sampling strategy was utilised to include a range of health professionals from a variety of different professions, levels of seniority, and genders. Once approval was received from managers, the lead researcher attended staff meetings to promote the study and distribute Expression of Interest forms. A mutually convenient interview time was arranged with staff who expressed interest.

### Data collection

Forty semi-structured interviews by phone (2) or face-to-face in a private office space (38) were conducted individually across the four psychiatric inpatient units between March and December 2016. All interviews were conducted by the female lead researcher, (COD) who is a registered Psychologist and PhD Candidate. The lead researcher also participated in a qualitative research methods course prior to conducting the analysis. Questions covered the following: type of care provided to women with a history of sexual violence; how care is implemented, and how it is supported. Questions were pilot tested with allied health professionals not employed by the Health Service. Interviews ranged from 12 to 90 min (average of 40 min). Field notes were made before, during and after the interviews. All interviews were audio recorded and fully transcribed verbatim by a professional transcription service. Feedback on transcripts was offered to all participants, two participants provided comments and corrections on their transcripts.

### Data analysis

Thematic analysis was used in this study [24], underpinned by a feminist theoretical framework that foregrounds gender as being central to the experience of sexual violence against women. Analysis focused on understanding healthcare professionals’ experiences and perceptions in providing care for women in psychiatric inpatient units who are survivors of sexual violence. It also focused on how this care was implemented (the basis of a forthcoming second publication). An inductive method was utilised to code the data by the lead researcher (COD) and co-coded by co-author (LT), moving from descriptive codes to interpretative codes and finally overarching themes. Relevant statements were coded with ample context to avoid data fragmentation and decontextualisation [23]. A final coding framework was agreed upon collaboratively with co-researchers and applied to the entire dataset. A selection of transcripts and quotes were reviewed collectively by all co-authors (COD, LT, SF, KH) under each theme to ensure consensus on the development [24]. In undertaking this process, it became evident that the overarching themes developed during analysis were in fact distinct typologies of participant experiences of care delivery. When the data were reviewed again with a focus on participant demographics, it revealed that responses from particular types of participants tended to fall within the different typologies; this additional layer of analysis is also described in our findings. The software program, NVivo 11 (QSR International, 2015) was used to manage the data and aid with analysis.

### Ethical considerations

The study was approved by both the Human Research Ethics Committee (HREC) at [Participating health organisation] and the HREC at [Researchers’ university]. Participation was confidential, and managers were not informed about who participated. Informed consent was obtained via a Plain Language Statement. Participants were asked to sign and return a consent form prior to commencing an interview. No incentive was offered.

## Results

Forty health professionals participated, including 20 nursing staff, 10 allied health professionals, seven medical staff and three consumer/peer support staff. Twenty-seven participants identified as female and thirteen as male. Thirty-one staff worked full-time and nine part-time. Participants had varying levels of experience working in their current role within the acute mental health service, ranging from 3 months to 29 years. The age of participants varied from 21 to 64 years of age with a mean of 42.5 years. Staff predominantly identified as being of Australian nationality (77.5%). Other staff identified as New Zealander, Maori, Chilean, Ghanaian, British, Greek, Indian, Italian and Singaporean.

Three main typologies were developed based on dominant themes within the dataset: *Dismissing and denying; Acknowledging but unprepared; Empathising but despairing.* Each of these typologies is described below with supporting quotes to illustrate them in more detail.

### Dismissing and denying

Some staff expressed negative attitudes towards female consumers who were believed to be more difficult to care for than male consumers. There was some reluctance from these staff to consider working on women only wards. George (Manager) illustrated this by recounting recent discussions with his colleagues: *“You wouldn’t get many who would want to work in a female only unit, because of the issues it causes, and women are, females generally are felt to be harder to look after …*” *.* This negative bias toward female consumers extends further to those with a diagnosis of Borderline Personality Disorder. Camilla (Medical) described her Registrar telling her*, “People with borderline, everyone sort of just runs away because they’re so difficult to manage”.* This perspective was frustrating for Maryann (Allied Health). She expressed her irritation with colleagues’ negative attitudes, flippant comments and lack of understanding towards women with a diagnosis of Borderline Personality Disorder: “*I guess where I see lack of respect is the ward’s attitude to women who have a diagnosis of personality disorder. A woman with a personality disorder comes from most likely 99.9 percent of child sexual abuse”.*

These negative attitudes towards female consumers resulted in staff not taking sexual assault disclosures seriously, minimising or blaming consumers. Elise (Allied Health) reported that female consumers were at times blamed for the perceived overexaggerated response to sexual violence. *“It’s one of the other unwell people who’s done this to [a young woman] consumer because he’s so unwell, immediately afterward, the staff was describing he had kind of humped her, and she reacted with rage at that, but then staff were kind of blaming her a bit, saying her reaction was over the top”.* Health professionals justified their dismissive, minimising or blaming responses using reasons which included lack of information on the incident of sexual violence, fear of retraumatising and time limitations of the care provision within a psychiatric inpatient unit. Compartmentalising incidents of sexual violence as historic, ‘confusing’ or expected due to the woman working in the sex industry permitted staff to ignore and reassure themselves that *‘it’s not why she’s here’* (Paul, Medical). Another reason for staff to justify their avoidance was expressed as a fear of *“opening old wounds”* by Jason (Allied Health) and a perception that it is better to “*leave what happened in the past, in the past”,* by Jane (Nurse). Kate (Allied Health) referred to the brevity of admission as a valid justification for this perception: “*We’re an inpatient unit, the average time is maybe a week that someone’s here. So, to reopen old wounds and to explore that, I don’t think it benefits them and is better for an outsourced service”.*

Additionally, some staff shied away from asking about sexual violence as they didn’t see it as part of their role. This view was particularly prominent for male staff and it was seen as a more appropriate role for female nurses. Jason (Allied Health) stated *“I guess my role isn’t sort of specified towards that”*. Ryan (Nurse) reported that providing care to survivors of sexual violence can be difficult for male staff “*if someone has that sexual abuse history, they just see every male as being the perpetrator and being someone that’s wanting to get them. That’s always a challenge, it can always be confronting”.* A male doctor, Paul, described nurses as the preferred staff for this role as they have more time to spend with patients than doctors. Nurses were considered to be intermediaries for disclosures of sexual violence from consumers who could then liaise with medical staff: “*… the next best person would be the nurse, because nurses will spend way more time with them. I would say on average statistically the patients might like nurses better than doctors, so they might get a bit more history, and they’ll flag it in their handover and it will be brought up to us as well”* (Paul, Medical). Patrick (Medical) reinforced this perception of providing care to women who are survivors of sexual violence as a nurses’ role *“I think general nursing staff who are more established are reasonably good”.* Ruth, (Medical) pointed out the limitations of relying on nursing staff only to provide this care *“the need for one-on-one staff we have to have nurses available to do that. One on one is pretty resource intense.”*

This division and avoidance was also reportedly evident at a leadership and an organisational level. Kaye (Allied Health) described, *“When we start talking about making definite decisions that affect everybody, the men are really engaged but when it’s considered almost secret woman’s business, they’re nowhere to be found. They’re not interested in the safety of their units, they’re not really interested in family violence”.* Furthermore, Matthew (Manager) expressed the view that desensitising consumers who had survived trauma was more important than ensuring their sense of safety and security on the ward: “*There was discussions about making one ward female only. No, because the world’s not like that, so it’s a fake sense of security … If you put them on a female ward then it’s gonna be a fake environment, because the world is not segregated. It’s about learning to manage. I know, trauma, and all that, but if we can learn to desensitise in the right way, that would help*”. These perceptions highlight the lack of understanding and reluctance of some health professionals to address women’s experiences of sexual violence.

### Acknowledging but unprepared

This theme describes the experience of many health professionals who recognised the prevalence and impact of sexual violence but felt unprepared to respond accordingly. Chris (Nurse) illustrates this understanding through his comment *“I think it [sexual violence] is a very common thing that’s for sure. A lot of our consumers who have borderline personalities tend to have a history of sexual assault, physical assault, mental even, so there have been numerous cases in the past”.* These staff also had good insight into how consumers may be feeling during their inpatient stay, as illustrated by Josie’s (Allied Health) comment: *“Essentially, it’s about not feeling safe and not being able to predict what might happen next, and a vulnerability about not necessarily having the capacity to make oneself safe”.* Incidents of sexual violence were reported by several healthcare professionals to have occurred on the psychiatric inpatient wards. These incidents, understandably, re-traumatised and negatively impacted a consumer’s recovery and ability to engage in treatment. This fear of harm while in the inpatient unit was real and a lived experience for female consumers as highlighted by Caroline’s (Nurse) comment: *“A male exposed himself to her [consumer] and was offering sexual favours and was blowing her kisses from a bedroom and he was taken out of the unit. It was a trigger for her because she had had an experience in the past where she was sexually abused. She struggled with her progression of her treatment here because it had brought up so much for her and she was really afraid and frightened. It was really hard to nurse her after that exposure and that experience”.*

In particular, many junior doctors and new graduate nursing and allied health professionals recognised that they might not have the skills to ask about histories of sexual violence and respond to disclosures. This was clearly reported by Camilla (Medical) *“We were never taught it in Med [ical] School, even in orientation. Here it would be useful to have a rundown of how to approach it. I ask the question quickly and then kind of move on”.* Elise (Allied Health) was frequently approached by junior medical staff asking for help to discuss trauma and sexual violence: *“I think a lot of staff here try hard to do their best. A lot of staff openly say they don’t quite know what to do. I’ve had doctors say to me they don’t know how to ask”.* Amanda (Nurse) declared her lack of knowledge in this area and desire to know more: *“I would like to know if there’s more that can be done. I guess for me, it’s a case of ‘I don’t know what I don’t know’. But I think there must be more things that we could do but I don’t know what they are”.* Furthermore, Ray (Allied Health) described his feelings of fear and inadequacy due to his lack of experience and limited time in his role: “*There are days when we don’t really feel prepared. There are days when I question whether or not I am ready or able, or capable of providing good service to [survivors of sexual violence]*”. Senior staff expressed concern as to how this affected appropriate follow-up, referrals, and care for female consumers within the inpatient unit: *“Well, everything is quite new to [graduate staff] so they’re not sure what to expect when I’ve asked them questions about their role and why things aren’t done, you know, this way for example. ‘We didn’t know’. And that’s understandable, but I’m just worried about how that then flows on and what they think about their roles and how that then influences our consumers”* (Madeline, Allied Health).

A level of confidence was noted by some senior female nursing staff in listening to women’s histories of sexual violence; however, this was often where their skills and confidence ended: *“I guess I suppose just being very, very conscious and very aware of it. If women want to talk to me about it, I’m more than happy to listen”* (Caroline, Nurse). Referrals to external agencies or to experienced allied health staff were perceived as the only avenues for care from this point for nursing staff. Olive (Nurse) describes: *“Again, I don’t know that we feel, or me particularly, or when people come to me to discuss it that we actually feel that we’re [in] the best position to talk about what’s happened in the past. We’ll happily discuss it, but how do we work around it or with it? There’s often referrals to other agencies or our social worker”.* However, senior male nurse Keith admits that seniority does not necessarily bring confidence or skills in caring for women who are survivors of sexual violence *“I don’t feel that I would be the best person to deal with difficult and very distressing issues for some people. I probably wouldn’t feel equipped or all that comfortable to deal with quite serious issues*”.

### Empathising but despairing

This theme encapsulates responses of several health professionals who understood the bidirectionality of sexual violence and mental illness and the individual responses from consumers. They expressed frustration towards colleagues and the mental health system for not responding sensitively or appropriately. Lucy (Nurse) described the individual experiences of sexual violence and the need for all staff to have an awareness of the possible impacts on victim-survivors. “*It [the impact of sexual violence] is completely subjective to the client. Just because you don’t feel that that is extreme, that’s your opinion. It is not how you approach in a hospital, or in any other setting. Especially with sexual assault. I think some people still need to grasp that”*. In addition, staff who understood the aetiology of Borderline Personality Disorder recognised the high prevalence of trauma and sexual violence these women have likely experienced. James (Manager) recognised that sexual violence is *‘a complex topic’* and that a staff member’s personal bias may influence the consumer’s decision to make a report: *“Often guilt is associated with the assault or abuse, working through that with the consumer, trying to be supportive of whatever decision they make; whether they want to report it or not. Trying to have their best interests in mind, but also respecting their choices regardless of what they may be and setting your own opinions aside and trying to remain supportive but neutral”.*

Empathising and believing women’s disclosures was more important to some staff members than others. These healthcare professionals were often frustrated with their colleagues who questioned the veracity of a disclosure of sexual violence due to psychosis or lack of evidence. Lucy (Nurse) empathetically discussed her understanding of psychotic symptoms as grounded in lived experience: *“I suppose instead of just pushing that aside as, ‘Oh, it’s just the psychosis’. You’re right, they are psychotic, but the memories of their experience from before is still real”*. Similarly, Angela (Allied Health) took the stance of believing the woman’s disclosure: *“Possibly [an allegation of sexual violence is] not true, but why did we believe it’s not true until we find out. I find that common here”*. Lauren (Nurse) explained this is an issue they frequently face within the unit and try to address through training and challenging perspectives: “*We’ll often get people, and they will report ‘I’ve been assaulted on the unit’. Sometimes it’s difficult for staff, they’ll think, ‘Oh, is it just part of a delusion or something like that?’ That’s a pretty big challenge, and a big part of the training as well. Where I try to emphasise that it [truthfulness] doesn’t matter”.* Madeline (Allied Health) described her strategy of acknowledging that their role is not to question but to create a safe and trusting environment for women to make disclosures: *“Unfortunately, I still get responses like, ‘We’re not sure if it’s true’, or ‘It’s part of her illness’. So really just making sure that it’s not our job to investigate whether something is true or not but more so to make sure that the person feels safe and has the option to disclose if they wanted to”.*

Staff were at times critical of the treatment recommended and provided to women. Some health professionals expressed their despair that certain retraumatising practices were still being carried out within the organisation. They had difficulty making decisions, asserting their opinions or having to ‘follow certain rules’ feeling it was incongruous with their personal beliefs and clinical judgement. Brooke (Nurse) illustrated this with a distressing example*: “My old nurse in charge, when the female nurses were off, organised male staff and male security staff to hold down and give a female consumer IM [intra muscular] injections. And that’s not on, so, that will be followed up by management because that’s just appalling behaviour and that shouldn’t be happening here. And it happened again last month. I’m horrified by the whole scenario. We’ve just traumatised a patient”.* Encouragingly some staff felt confident enough to raise their concerns with management; however, this is not always the case. A barrier to assertively raising their concerns was a lack of confidence to speak up and disagree with more dominant colleagues as noted by Charlotte (Manager): *“I think sometimes it’s about confidence, that sometimes a colleague is a lot stronger in verbalizing what they think should go on, or even sometimes if I [the manager] go in, I notice that people withdraw. They’ll wait for that authority, for people to decide for them. We’re really trying to change this”.* In these instances, staff sought out and relied on management to assert their authority rather than feeling that they could be assertive or raise their concern themselves.

The main findings are summarised below in Table 1. The typology of *dismissing and denying* consisted predominantly of male staff who expressed fearfulness and ambivalence towards female consumers. The typology of *acknowledging but unprepared* consisted of male and female staff who expressed anxiety and a lack of confidence in response to providing care to survivors of sexual violence. The third typology, *empathising but despairing,* denotes health professionals’ frustration with their colleagues and a sense of powerlessness with leadership and the health system.

**Table 1:** Typology summary table

Theme	Emotional Experience	Occupation	Gender	Position level	Years of experience in current setting
Dismissing and Denying	Fear and ambivalence	Medical, Allied Health & Nurses	Mainly male staff & comments by female staff about colleagues	Graduate and junior staff but also senior management staff	Mainly less than 2 years’ experience, some between 4 and 6 years’ experience, one staff with more than 10 years’ experience
Acknowledging but unprepared	Anxiety and lack confidence	Medical, Allied Health & Nurses	Male and female	Graduate and junior staff, some senior nursing staff	Half with less than 2 years’ experience and half with more than 10 years’ experience
Empathising but despairing	Frustration and powerlessness	Nurses & Allied Health	Mainly female staff	Senior to junior staff	Mainly less than 2 years’ experience, some with 5 years’ experience, some staff with more than 10 years’ experience.

## Discussion

This study explored Australian health professionals’ perceptions of care provided for women in psychiatric inpatient settings who are survivors of sexual violence.

Our findings suggest that health professionals working in the participating psychiatric inpatient units fell into three groups: those who expressed disparaging perceptions towards female consumers resulting in minimising, ignoring or blaming responses to disclosures of sexual assault; those who recognised the prevalence of sexual violence but felt unequipped to respond appropriately; and those who understood the complex bidirectionality and the limitations of the care provided. Attitudes within these groups were mostly associated with particular types of participants, suggesting that training around gender-sensitive care may need to be targeted based on role, gender and years of experience.

The disparaging perceptions of female consumers in this study were predominantly held by male staff and less experienced health professionals. Health professionals who lack experience have been found more likely to be disrespectful or inconsiderate of survivors [25]. Unfortunately, negative responses, such as victim blaming, doubting the veracity of a story, minimising the seriousness of the violence or denying assistance, are not uncommon from health professionals, particularly men [26]. Specifically, consistent with the findings of this study, negative attitudes are often reported towards consumers with a diagnosis of Borderline Personality Disorder [27]. Such responses result in less empathy and retraumatisation of survivors [26].

Staff dismissed and ignored disclosures of sexual violence in a number of ways. Sexual violence is often overlooked as secondary to a diagnosed psychiatric disorder, dismissed as a symptom of psychosis or minimised as historical, therefore not relevant to the consumer’s current presentation [28, 29]. These practices further contribute to the re-victimisation of consumers, in addition to the witnessed and experienced traumatic events that occur during admission to a psychiatric inpatient unit [29, 30]. Furthermore, the use of ‘alleged’ when recording disclosures on medical notes is another subtle form of dismissing a disclosure of sexual violence [31]. Health professionals relate these practices to the limited time they have with consumers on psychiatric inpatient units [13]. Ultimately, such attitudes and practices of health professionals continue to silence and re-victimise consumers [21].

Male health professionals in this study distanced themselves from providing care for women who are survivors of sexual violence, believing it to be a more appropriate role for a female nurse. This is consistent with previous believes held by health professionals [32]. However, this is contrary to reports from victim/survivors, that gender of the clinician is irrelevant when they provide a supportive response to a disclosure of violence [33]. It is important to be cognisant of the sociocultural norms that reinforce attitudes of violence against women that might be shared by health professionals [34].

Staff acknowledged that sexual violence was a serious issue for many of the female inpatients. Nonetheless, nurses and allied health staff in this study reported feeling anxious, frustrated and powerless to respond appropriately to consumers with such experiences. This may also be a result of the dominant biomedical model that typically informs the mental health sector in Australia [35] despite the presence of gender-sensitive and trauma-informed policies in place across the participating psychiatric units. The biomedical model trains health professionals to focus on diagnosis, treatment, and prescription of medication [17], rather than how to respond to the impacts of trauma, with trauma symptoms seen as pathological rather than adaptive [31, 36]. This narrow focus results in a lack of adequate training on sensitive inquiry and response to disclosure of sexual violence. Thereby perpetuating a cycle of anxiety, avoidance and the use re-traumatising practices such as restrictive interventions, body searches and round the clock observations [21, 22, 37]. This approach is counterproductive for consumers and can result in vicarious trauma for health professionals [38, 39].

Consistent with previous research [40], our study suggests that female health professionals, who were predominately nurses and allied health staff, not only have greater empathy for female consumers, but also have greater understanding of sexual violence than their male or less experienced colleagues. They are also more likely to assess for trauma than their male colleagues [14]. Health professionals expressed frustration due to the disparaging perceptions held by their colleagues and the focus on symptomatology of the biomedical model. These challenges were further compounded by the hierarchy amongst health professionals working in this setting and the difficulty speaking up against the system [41]. To overcome this challenge, meaningful collaboration with other nurses and consumers provides an alternative but cohesive way of functioning [41]. Health professionals and management need to understand these power dynamics that are often enacted within acute psychiatric inpatient units.

Trauma-informed care is a model that offers a solution to these challenges in mental health settings. It provides an opportunity for improved experiences and relationships between consumers and health professionals through greater understanding, respect and trust [22]. This model of care sees the consumer within a holistic context and it acknowledges the pervasiveness, directionality and impact of trauma [42–44]. Considering the above typologies within the trauma-informed care framework, the *dismissing and denying* group could be considered ‘trauma-uninformed’ [22] due to their negative attitudes towards female consumers and dismissiveness towards histories of sexual violence. The *acknowledging but unprepared* group, display emerging trauma-informed skills, as they demonstrate an understanding of the extent and impacts of sexual violence on a consumer’s current wellbeing and feelings of safety but lack the skills and competencies to implement into their practice. The *empathising but despairing* group appear to have a fuller understanding of adopting a trauma-informed care approach, in addition to the skills and competencies to implement this in their practice. Understanding health professionals’ level of knowledge of trauma-informed care, could help tailor training to meet their individual needs.

### Limitations

Although participants were recruited from four different psychiatric inpatient units, these were all within the one mental health organisation. This sample is likely to have been informed by health professionals who had a greater knowledge of gender-sensitive issues than other mental health professionals. This sample comprising two-thirds females and half nurses, is representative of the female and nursing dominated mental health workforce [45]. Researcher bias was minimised through co-development of the coding framework and collaborative review of themes between four members of the research team (each drawing on different disciplinary backgrounds). However, it is still possible that the experiences of the research team influenced our interpretation of data.

### Implications

Despite numerous training packages being implemented within mental health settings, disparaging perceptions or avoidance of providing care for survivors of sexual violence continues. There is some evidence to show that education and training on the effects of trauma and challenging paternalistic views has positive effects in changing attitudes of doctors and nurses [46]. Training programmes need to be tailored to health professionals’ level of knowledge, their environment and provide clinical skills to sensitively identify, discuss and respond to trauma and sexual violence [14, 47]. Health professionals should also be encouraged to discuss and reflect on their personal gender stereotypes that perpetuate these pejorative perceptions and adversely impact on the care provided [35]. Further research is needed in this area. Policies need to clearly prioritise and address gender and sexual violence. Importantly, policy and practice need to align. Successful implementation of policy is dependent on health professional attitudes, knowledge and preparedness to respond to trauma [14]. However, trauma-informed care is more than its sum of its parts; principles, understanding of trauma, reflective practice, individual practices or policy [48]. Comprehensive implementation is complex, multi-layered and requires whole of organisation approach over a sustained period of time [48, 49].

## Conclusion

These findings highlight the presence of disparaging perceptions towards female consumers who are survivors of sexual violence and thereby the impact this has on the care they receive in psychiatric inpatient units. To date, there has been limited qualitative research into health professionals’ perceptions of care provided in this setting. This is problematic as it has overlooked the tension between the collective attitudes held by health professionals depending on their training, gender and years of experience. Most research investigating mental health and trauma has centred around the reduction of restrictive interventions and seclusion, practical strategies of implementing trauma informed care, changes to leadership-styles, modification to the historical authoritarian nurse-consumer relationships and changes to policies [35]. Further research is needed to understand origins of health professionals’ perceptions and ways of changing perceptions, attitudes and culture.

## Data Availability

The datasets used and/or analysed during the current study are available from the corresponding author on reasonable request.
